# A 3-Week Multidisciplinary Body Weight Reduction Program Improves Body Composition and Lower Limb Power Output in 3,778 Severely Obese Children and Adolescents

**DOI:** 10.3389/fphys.2020.00548

**Published:** 2020-05-28

**Authors:** Stefano Lazzer, Giulia Bravo, Gabriella Tringali, Roberta De Micheli, Alessandra De Col, Alessandro Sartorio

**Affiliations:** ^1^Department of Medicine, University of Udine, Udine, Italy; ^2^School of Sport Science, University of Udine, Udine, Italy; ^3^Istituto Auxologico Italiano, Istituto di Ricovero e Cura a Carattere Scientifico (IRCCS), Experimental Laboratory for Auxo-endocrinological Research, Verbania, Italy; ^4^Istituto Auxologico Italiano, Istituto di Ricovero e Cura a Carattere Scientifico, Division of Auxology & Metabolic Diseases, Verbania, Italy

**Keywords:** muscular anaerobic power, body composition, bioelectric impedance analysis, childhood & adolescence, physical activities and sports

## Abstract

The aim of the present study was to investigate the effects of a 3-week in-hospital body weight reduction program (BWRP), entailing moderate energy restriction, physical activity, psychological counseling and nutritional education, on body composition and lower limb muscle power (LLP) output in obese children and adolescents. Three thousand seven hundred seventy-eight obese [BMI: 36.2 ± 5.9 kg⋅m^–2^; fat mass (FM): 42.7 ± 4.0%] children and adolescents (2,318 girls and 1,460 boys, aged 8–18 year) participated in this study. Before (T0) and after the end of the BWRP (21st day, T21), body composition was assessed by an impedancemeter and LLP by the Margaria stair climbing test. Body mass (BM) and FM significantly decreased in girls (-4.8 and –7.1%, *p* < 0.001) and in boys (-5.5 and -9.3%, *p* < 0.001) after 3-week BWRP, while fat-free mass (FFM) did not change significantly in both genders. LLP expressed in absolute values (W) significantly increased in girls (by mean 6.4% from age 13 to 18 year, *P* < 0.001) and in boys (by mean 7.2% from age 12 to 18 year, *P* < 0.001). LLP normalized to BM (W⋅kg^–1^BM) significantly increased in girls (by mean 11.3%, *P* < 0.001) and boys (by mean 12.6%, *P* < 0.001) from age 9 to 18 year. As well, LLP normalized to FFM (W⋅kg^–1^FFM) significantly increased in girls (by mean 9.1% from age 9 to 18 year, *P* < 0.001) and in boys (by mean 10.1% from age 10 to 18 year, *P* < 0.001). In conclusion, 3-week BWRP induces a significant decrease in FM and maintenance in FFM in obese children and adolescents, these effects being also associated with a significant increase of LLP both in absolute terms and when normalized to the BM or FFM.

## Introduction

Childhood obesity rates and the co-morbidities of obesity are rising throughout the industrialized countries (Cattaneo et al., 2010). The most significant health consequences related to childhood obesity include hypertension, type 2 diabetes, cardiovascular disease, certain types of cancer and psychosocial problems (Cote et al., 2013). Effective weight-management programs, in children and adolescents, involves multiple techniques and strategies including dietary therapy, physical activity and behavior therapy to foster long-term weight control and prevention of weight regain. While dietary therapy has shown a greater weight loss than physical activity alone (Kiortsis et al., 1999), increased physical activity is not only important for weight loss (Swinburn and Ravussin, 1993) and weight loss maintenance (van Baak et al., 2003) but also have positive impact on metabolic and cardiovascular risk profiles (Roberts et al., 2015).

Physical activity relies on aerobic and anaerobic energy transfer processes. While aerobic power provides energy needed for moderate-intensity and prolonged activities, anaerobic power provides energy needed for rapid horizontal acceleration and vertical lifting of the body mass, as those commonly performed in everyday life and leisure time activities. Previous studies have shown that obese children and adolescents had lower aerobic and anaerobic capacities to a various degree than their normal weight counterparts (Loftin et al., 2001; Torok et al., 2001; Gidding et al., 2004; Thivel et al., 2011). The stair climbing test (SCT) modified from the test proposed by Margaria et al. (1966) has been applied in severely obese children and adolescents (Maffiuletti et al., 2004; Sartorio et al., 2006), adults (Sartorio et al., 2001, 2003b,c, 2004c, 2005; Lafortuna et al., 2002, 2004, 2005), and elderly (Sartorio et al., 2004a) to evaluate anaerobic working capacity, particularly maximal lower limb muscle power. In obese children and adolescents (Maffiuletti et al., 2004; Sartorio et al., 2006) and adults (Sartorio et al., 2001, 2003b,c, 2004c, 2005; Lafortuna et al., 2002, 2004, 2005), maximal lower limb muscle power output widely varies with age, body mass index (BMI) and gender, with a major determinant of these power variations being a concomitant variation in body composition (Lafortuna et al., 2005; Sartorio et al., 2006). Through the ages of life from childhood to adolescence, body composition varies greatly with different trends in boys and girls (Malina et al., 1995; Grund et al., 2000; Lazzer et al., 2003; Wells et al., 2012), although entailing consequences on muscle power generation (Sartorio et al., 2006). In addition, childhood obesity interferes with maturational processes around puberty (Garn and Clark, 1975; Shalitin and Kiess, 2017), and affects muscle machinery and then physical capacities.

Thus, the aim of the present study was to determine whether a short-term (3-week) multidisciplinary body weight-reduction program (BWRP) in a specialized institution, entailing physical activity, moderate energy restriction, psychological counseling and nutritional education, can affect changes in body composition and lower limb muscle power in severely obese children and adolescents.

## Materials and Methods

### Subjects

Three thousand-seven hundred seventy-eight severely obese children and adolescents (2,318 girls and 1,460 boys; age range 8–18 year) participated in this study [about 300 of the 3,778 children and adolescents included in the present study had participated in a previously published study (Maffiuletti et al., 2004; Sartorio et al., 2006)]. The BMIs for gender and chronological age were above the 99th percentile (Cole et al., 2000). The subjects were recruited as in-patients from the Division of Auxology, Italian Institute for Auxology, IRCCS, Piancavallo (VB) Italy. None had been involved in structured physical activity (i.e., regular activity more than 60 min⋅week^–1^) before the study. All subjects had a full medical history and physical examination, with the routine hematology and biochemistry screens and urine analysis. None of the children had signs or symptoms referable to major cardiovascular, respiratory or orthopedic disease contraindicating or interfering significantly with the performance of the motor test used in the study. By the time of the measurements, all the subjects were familiar with the experimental protocol.

### Study Protocol

The study was approved by the Ethics Committee of the Italian Institute for Auxology (Milan, Italy; research project code: 01C824; acronym: POTARTINFOB) and was in accordance with the Helsinki Declaration of 1975, as revised in 2008. The purpose and objective of the study were explained to each subject and his/her parents, and written informed consent was obtained before beginning the study. The patients were hospitalized for a period of 4 weeks at the Division of Auxology, Istituto Auxologico Italiano, IRCCS, Piancavallo (VB). During the first 3–4 days subjects underwent physical examination, routine hematology and biochemistry screens and urine analysis. Thereafter, they followed a 3-week personalized BWRP consisting of lifestyle and dietary education, physical activity and psychological counseling. Full testing sessions were carried out just before the beginning (baseline, T0) and at completion of the 3-week BWRP (T21). The testing session included assessment of anthropometric characteristics, body composition and lower limb muscle power (see below for the detailed description).

### Body Weight-Reduction Program (BWRP)

#### Diet and Nutritional Education

During the 3-week BWRP, personalized diets were offered on the basis of the initial basal metabolic rate test and physical activity level for each young patient. The calories to be introduced with the diet were calculated by subtracting ~25% from the value of resting energy expenditure as measured in each patient by indirect calorimetry (Vmax 29; SensorMedics Corporation, Yorba Linda, CA, United States) for a total duration of 20 min. Energy supply containing 21% proteins, 53% carbohydrates, and 26% lipids. The composition of diet was formulated according to the Italian recommended daily allowances (Nutrition ISo, 1996). Each patient was free to choose foods from a heterogeneous daily menu. Foods to which the patient declared to be allergic were removed from the menu. Five daily portions of fruits and vegetables were mandatory. A fluid intake of at least 1,500 mL⋅day^–1^ was encouraged. Moreover, the dieticians’ team verified that each subject had finished every meal (all the subjects considered in the present study finished the 98% of the meals). During the BWRP the patients had dietetics lessons consisting of lectures, demonstrations and group discussions with and without a supervisor, which took place every day throughout the whole rehabilitation period.

#### Psychological Counseling

Psychological sessions were led by clinical psychologist 2–3 times per week and were based on cognitive-behavioral strategies with individual or group sessions, e.g., stimulus control procedures, problem solving and stress management training, development of healthy eating habits assertiveness a social skills training, cognitive restructuring of negative maladaptive thoughts and relapse prevention training. When possible (1 day per week), supplementary sessions were also performed with patients’ parents, aimed at improving motivation in changing lifestyle and interpersonal communication.

#### Physical Activity

During the 3-week BWRP, children and adolescents participated in a personalized exercise-training program, from Monday to Friday, under the guidance of a therapist. Training sessions lasted 45–60 min per day (preceded and followed by 5–7 min stretching) and were mainly made up of aerobic activities (walking on a treadmill or cycling on an ergometer) under heart rate (HR) monitoring and medical supervision. All subjects completed 15 sessions of physical training. The intensity of endurance exercises was set at a HR corresponding to 60 and 80% of the individual maximal HR estimated as 220-age (year).

In addition, subjects had 1 h/day of aerobic leisure activities at the institution on Saturday and Sunday. The research assistant and the physical trainers verified that each subject participated in each training session, performed exercises correctly, and completed at least 95% of the exercise session and program. Additionally, all the subjects considered in the present study completed more than 96% of the exercise session and program.

## Measurements

### Physical Characteristics and Body Composition

The medical history and a physical examination of subjects were taken at the time of admission to hospital. Body mass (BM), evaluated at the admission to the hospital, at T0 and T21, was measured to the nearest 0.1 kg with an electronic scale (Selus, Italy) with the subject wearing only light underclothes. Stature was measured to the nearest 0.5 cm on a standardized Harpenden stadiometer (Holtain Ltd., United Kingdom). The body mass index (BMI) was calculated as weight (kg) divided by stature (m) squared. The standard deviation score (SDS) of BMI-SDS was calculated applying the LMS method (Cole et al., 1998) to Italian reference values for children and adolescents (Cacciari et al., 2002). Body composition was measured at T0 and T21 by using a multifrequency tetrapolar impedancemeter (BIA, Human-IM Scan, DS-Medigroup, Milan, Italy) with a delivered current of 800 μA at a frequency of 50 kHz. In order to reduce errors of measurement, attention was paid to the standardization of the variables that affect measurement validity, reproducibility, and precision. Measurements were performed according to the method of Lukaski (1987) (after 20 min resting in a supine position with arms and legs relaxed and not in contact with other body parts) and in strictly controlled conditions in accordance with the NIH guidelines (NIHT, 1996). As well, the menstrual cycle phase has been considered because it could actually influence fluid retention and consequently the measurement of body composition by BIA. In post-menarcheal girls, we did not take the BIA measurements during menses, as suggested by the NIH guidelines. FFM was calculated using the prediction equation developed by our group (Lazzer et al., 2008), and fat mass (FM) was derived as the difference between BM and FFM. The equation developed by our group (Lazzer et al., 2008) considered similar population than in the present study, but none of the subject involved in the present study were used in our previous study. Finally, fat mass index (FMI) was determined by dividing FM (kg) by stature squared (m). Similarly, fat-free mass index (FFMI) was calculated by dividing FFM (kg) by stature squared (m), as proposed by VanItallie et al. (1990).

### Lower Limb Muscle Power

Stair climbing test (SCT), which allows measuring the maximal anaerobic power of the involved muscles, was performed at T0 and T21 of BWRP. SCT consists in a modification of the test proposed by Margaria et al. (1966) hat has been applied in severely obese children (Maffiuletti et al., 2004; Sartorio et al., 2006) and adults (Sartorio et al., 2001, 2003b,c, 2004c, 2005; Lafortuna et al., 2002, 2004, 2005). At the moment of the first execution, 2–3 practice trials were allowed so that the subjects gained a good control of the performing technique. Subjects were asked to climb up ordinary stairs at the highest possible speed, according to their capabilities, by using every second step. The stairs consisted of 13 steps of 15.3 cm each, thus covering a total vertical distance of 1.99 m. An experimenter measured the time employed to cover the test with a digital stopwatch. In line with Margaria et al. (1966) assumption, muscle power was calculated by using the following equation:

SCT power (W⋅kg^–1^) = (9.81 ⋅ vertical distance) ⋅ time^–1^

where vertical distance (i.e., 1.99 m) and time are expressed in m and s, respectively, and 9.81 m⋅s^–2^ represents the acceleration of gravity. SCT repeatability in adults obese individuals has been previously assessed in our laboratory and the coefficient of variation between measurements has been found to be lower than 5% (Sartorio et al., 2001).

## Statistical Analyses

Statistical analyses were performed using by SAS, Release 9.4 (SAS Institute, Cary, NC, United States), with a significance set at *P* < 0.05. All results were expressed as means and standard deviation (SD). Normal distribution of the data was tested using the Kolmogorov-Smirnov test.

The effects of gender, age and time categories and interaction between these variables on physical characteristics, body composition and lower limb muscle power, were tested using General Linear Model repeated measures, after evaluating also the homogeneity of variance with Levene test.

When significant differences were found, a Bonferroni *post hoc* test was evaluated implementing multiple comparison.

## Results

### Physical Characteristics and Body Composition

**FIGURE 1:**
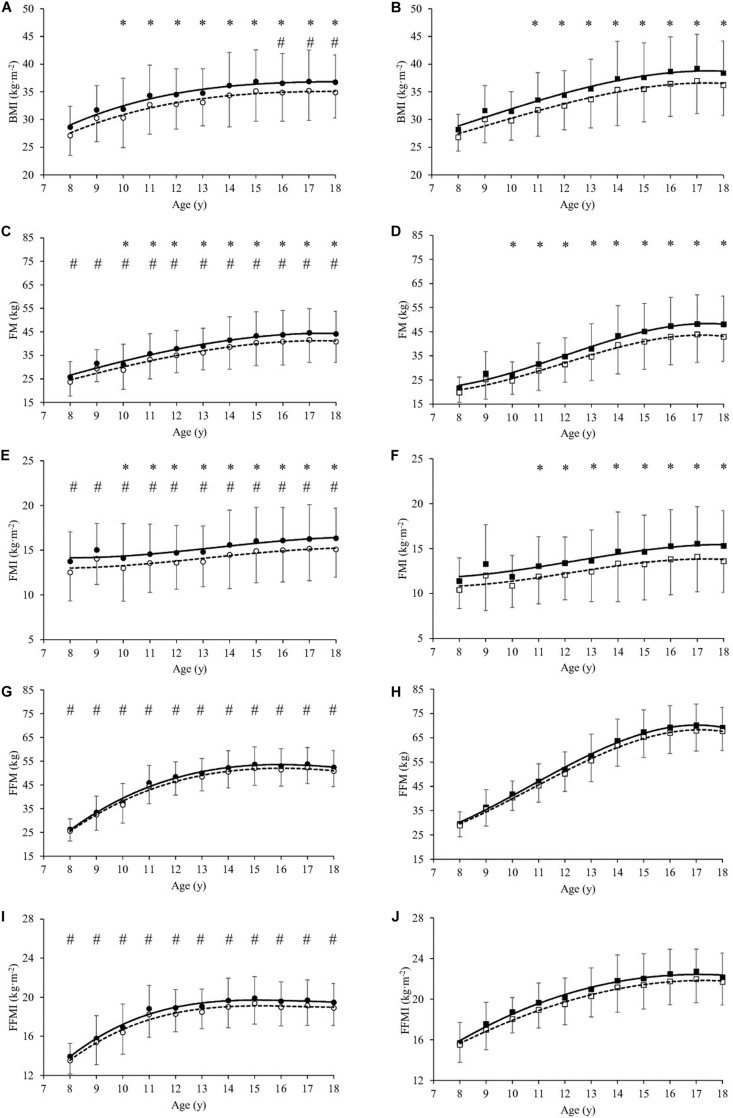
Body Mass Index (BMI in kg⋅m^–2^, **A,B**), Fat Mass (FM in kg, **C,D**), Fat Mass Index (FMI in kg⋅m^–2^, **E,F**), Fat-Free Mass (FFM in kg, **G,H**) and Fat-Free Mass Index (FFMI in kg⋅m^–2^, **I,J**) as a function of age (year) in girls **(A,C,E,G,I)** and boys **(B,D,F,H,J)**, before (T0, filled symbols) and at the end (T21, empty symbols) of the weight-management program. All values are mean and standard deviation (SD). Significance by General Linear Model repeated measures. *significantly different (*P* < 0.05) between T0 and T21 at same age. #significantly different (*P* < 0.05) between girls and boys at same age.

In basal condition (T0), significant differences were observed in the anthropometric and body composition characteristics between girls and boys (Table 1). Stature, BM and BMI-SDS were significantly lower in girls than boys by mean 3.3, 9.9, and 2.5%, respectively (*P* < 0.001, Table 1). Analysing the parameters across the entire age range, BMI values were significantly lower in girls than boys by mean 5.5% from age 16 to 18 year (Figures 1A,B), FM was significantly higher in girls than boys by mean 10.3% from age 8 to 13 year (Figures 1C,D), while it was significantly lower by mean 6.8% from 14 to 18 year. FMI values were significantly higher in girls than boys by mean 9.1% from age 8 to 18 year (Figures 1E,F). FFM was significantly lower in girls than boys by mean 19.2% from age 8 to 18 year (Figures 1G,H). FFMI was significantly lower in girls than boys by mean 11.0% from age 8 to 18 year (Figures 1E,F).

**TABLE 1:** Physical characteristics of children and adolescents before (T0) and after (T21) of the body weight-reduction program.

	**Girls (*n*: 2318)**		**Boys (*n*: 1460)**		***P***		
	**T0**	**T21**	**T0**	**T21**	**G**	**T**	**G × T**
Stature (m)	1.60 ± 0.08		1.67 ± 0.12		0.001		
BM (kg)	92.3 ± 17.8	88.0 ± 16.9	103.7 ± 24.6	97.9 ± 23.2	0.001	0.001	0.431
BMI (Kg⋅m^–2^)	35.8 ± 5.6	34.1 ± 5.3	36.8 ± 6.3	34.7 ± 6.0	0.001	0.001	0.526
BMI (SDS)	2.81 ± 0.53	2.63 ± 0.55	2.93 ± 0.64	2.70 ± 0.66	0.001	0.001	0.254
FFM (kg)	51.0 ± 8.5	49.5 ± 8.2	61.8 ± 13.0	59.9 ± 12.5	0.001	0.085	0.497
FFMI (kg⋅m^–2^)	19.3 ± 2.3	18.7 ± 2.1	21.4 ± 2.7	20.8 ± 2.6	0.001	0.092	0.254
FM (kg)	41.4 ± 10.3	38.5 ± 9.7	41.9 ± 12.9	38.0 ± 12.1	0.969	0.001	0.032
FMI (kg⋅m^–2^)	15.6 ± 3.6	14.5 ± 3.4	14.4 ± 3.9	13.1 ± 3.8	0.001	0.001	0.102
FM (%)	44.5 ± 3.8	43.4 ± 3.8	39.9 ± 4.3	38.3 ± 4.6	0.001	0.001	0.148
SCT (s)	3.6 ± 0.6	3.2 ± 0.5	3.4 ± 0.7	3.0 ± 0.6	0.001	0.001	0.074
SCT (W)	514 ± 115	545 ± 123	614 ± 166	656 ± 181	0.001	0.001	0.514
SCT (W⋅kg^–1^ BM)	5.6 ± 0.9	6.2 ± 1.0	6.0 ± 1.1	6.7 ± 1.2	0.001	0.001	0.487
SCT (W⋅kg^–1^ FFM)	10.1 ± 1.6	11.0 ± 1.7	9.9 ± 1.7	10.9 ± 1.9	0.065	0.001	0.098

**All values are mean and standard deviation (SD). BMI, body mass index; BMI (SDS), body mass index standard deviation score; FFM, fat-free mass; FFMI, fat-free mass index; FM, fat mass; FMI, fat mass index; SCT, stair climbing test. Significance by General Linear Model repeated measures; Gender (G) and Time (T); Gender × time interaction (G × T).**

At the end of the BWRP (T21), BM and BMI-SDS significantly decreased in girls (by mean 4.8 and 6.5%, respectively, *P* < 0.001, Table 1) and in boys (by mean 5.5 and 7.4%, respectively, *P* < 0.001, Table 1). Analysing the parameters across the entire age range, BMI decreased significantly after BWRP in girls (by mean 4.8% from age 10 to 18 year, *P* < 0.001, Figure 1A) and boys (by mean 5.5% from age 11 to 18 year, *P* < 0.001, Figure 1B). FM decreased significantly in girls (by mean 7.1%, *P* < 0.001) and boys (by mean 9.3%, *P* < 0.001) from age 10 to 18 year (Figures 1C,D).

FMI decreased significantly in girls (by mean 7.2%, *P* < 0.001) and boys (by mean 9.5%, *P* < 0.001) from age 10 to 18 year (Figures 1E,F). A slight, but not significant, decrease in FFM was observed in girls and boys (Figure 1E,F); also, FFMI did not change significantly in girls and boys after BWRP (Figures 1I,J).

### Lower Limbs Muscle Power

**FIGURE 2:**
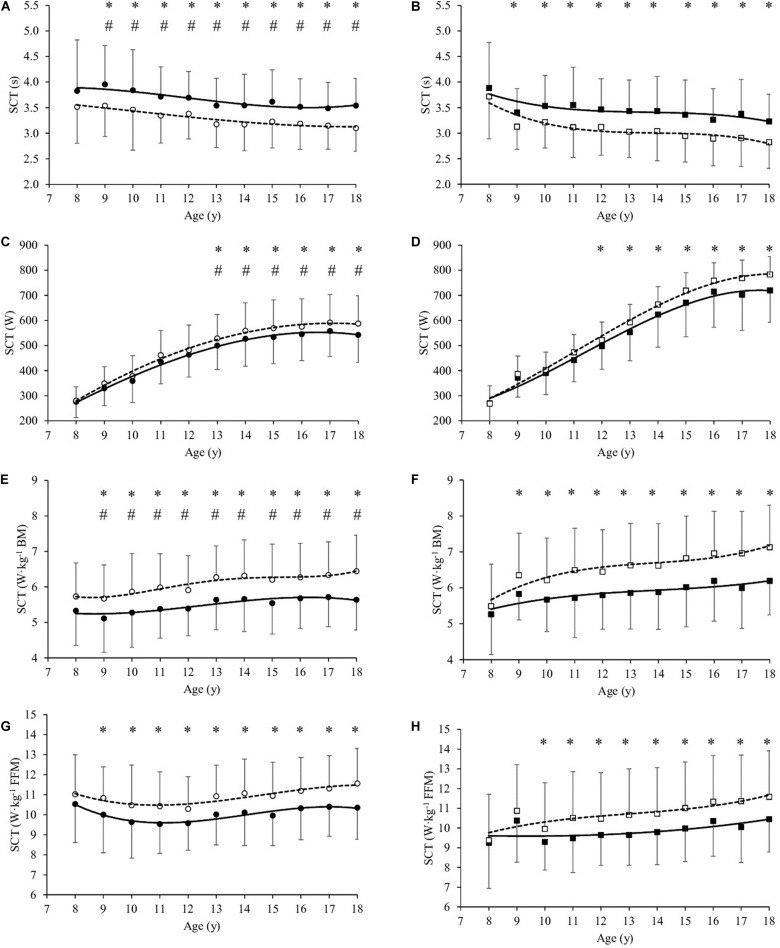
Stair climbing test values (SCT in s, **A,B**), SCT (W, **C,D**), SCT (W⋅kg^–1^ Body Mass, **E,F**) and SCT (W⋅kg^–1^ Fat-free Mass, **G,H**) as a function of age (year) in girls **(A,C,E)** and boys **(B,D,F,H)**, before (T0, filled symbols) and at the end (T21, empty symbols) of the body weight-reduction program. All values are mean and standard deviation (SD). Significance by General Linear Model repeated measures. *significantly different (*P* < 0.05) between T0 and T21 at same age. #significantly different (*P* < 0.05) between girls and boys at same age.

At baseline (T0), SCT in s was significantly higher in girls than boys by mean 6.6% from age 9 to 18 year (Table 1 and Figures 2A,B). SCT expressed in absolute values (W) was significantly lower in girls than boys by mean 24.3% (*P* < 0.001, Table 1) from age 13 to 18 year (Figures 2C,D). Similarly, SCT normalized to BM (W⋅kg^–1^ BM) was significantly lower in girls than boys by mean 7.5% (*P* < 0.001) from age 9 to 18 year (Table 1 and Figures 2E,F). By contrast, SCT normalized to FFM (W⋅kg^–1^ FFM) was not significantly different between girls and boys (Table 1 and Figures 2G,H).

SCT in s significantly decreased after BWRP (T21 vs. T0) in girls (by mean 10.2%, *P* < 0.001) and boys (by mean 11.2%, *P* < 0.001) from age 9 to 18 year (Figures 1A,B, respectively). SCT expressed in absolute values (W) significantly increased in girls (by mean 6.4% from age 13 to 18 year, *P* < 0.001, Figure 2C) and boys (by mean 7.2% from age 12 to 18 year, *P* < 0.001, Figure 2D). SCT normalized to BM (W⋅kg^–1^ BM) significantly increased in girls (by mean 11.3%, *P* < 0.001) and boys (by mean 12.6%, *P* < 0.001) from age 9 to 18 year (Figures 2E,F, respectively). SCT normalized to FFM (W⋅kg^–1^ FFM) significantly increased in girls (by mean 9.1% from age 9 to 18 year, *P* < 0.001, Figure 2G) and boys (by mean 10.1% from age 10 to 18 year, *P* < 0.001, Figure 2H).

## Discussion

The 3-week multidisciplinary BWRP entailing lifestyle education, moderate energy restriction, psychological counseling and physical activity resulted in: (1) decrease in BM, FM and FMI; (2) decrease in time of execution SCT in girls and boys, thus resulting in a significant muscle power increase estimated during SCT, both in absolute terms and normalized to the BM or FFM.

The main objectives of the BWRP are to reduce body weight, particularly FM and FMI, and encourage long term weight control and prevention of weight regain. Previous studies, in children and adolescents, showed that energy restriction without physical activity induces reduction in FM as well as in FFM (Zwiauer et al., 1992; Kiortsis et al., 1999; Tounian et al., 1999). However, when physical activity was or was not associated with an energy restriction, FFM was maintained while FM decreased (Sothern et al., 1999; Gutin et al., 2002). Preventing the decrease of FFM during a BWRP is a very important result, as the FFM is positively related to the physical capacities and the basal metabolic rate (Lazzer et al., 2010), which accounts for 60–70% of daily energy expenditure (Lazzer et al., 2003).

In the present study, moderate energy restriction and physical activity were capable to reduce FM and FMI and maintain FFM and FFMI, although a slight decrease in FFM was observed. Previous studies shown that including specific strength training in BWRP favor increase in FFM in obese children and adolescents (Stiegler and Cunliffe, 2006). These data are similar to those previously reported in our laboratory after 3-week BWRP for severely obese patients aged 8–17 year (Maffiuletti et al., 2004; Lazzer et al., 2011), 18–46 year (Sartorio et al., 2003a) and even for elderly obese male and females aged 61–75 year (Sartorio et al., 2004b), confirming that body composition of obese subjects could be improved at all ages.

In the present study, body composition was measured using BIA on the basis of water content in the body (Lukaski et al., 1986). All measurements of body composition in our study were performed under strictly controlled conditions in accordance with the NIH guidelines (NIHT, 1996). BIA is a common, simple, rapid, and non-invasive method to estimate total body water and FFM in healthy subjects as well as in obese subjects (Houtkooper et al., 1996). BIA has been cross validated in children and adolescents against measurements of total body water by deuterium dilution (Wabitsch et al., 1996) and total body potassium (Schaefer et al., 1994). The accuracy of BIA is highly dependent on the equations used to calculate FFM. The BIA prediction equations developed previously by our group against DEXA in obese children and adolescents (Lazzer et al., 2008), have been used in the present study to reduce inaccuracies in body composition measurements.

A modified Margaria test (Margaria et al., 1966) has previously used as method to evaluate lower limb muscle power in obese subjects (Lafortuna et al., 2002), and it has been clinically used to evaluate motor performance in response to body weight management programs (Sartorio et al., 2001, 2003b,c, 2004a,c, 2005, 2006; Lafortuna et al., 2002, 2004, 2005; Maffiuletti et al., 2004). In the obese children and adolescents of the present study, absolute voluntary lower limb muscle power was affected by gender and age. Particularly, absolute power increased with age in both genders, and girls and boys attained similar values up to the age of 12 year. Nonetheless, after the age of 12 year boys continued to attain an increasing global power output, while girls reached a plateau, their values being significantly lower than boys in this range of age (Figures 2C,D). This gender-related specific course during the period of puberty may indicate that it is influenced by hormonal changes. In addition, a trend similar to that observed by absolute power was also observed in the age-related rise of FFM, with boys after 12 year showing significantly higher FFM amounts than girls. This finding is in line with previous studies (Veldhuis et al., 2005; Sartorio et al., 2006), showing no gender difference in FFM prior to adolescence, with a tendency to plateau in girls at age 12–13 and a continuing increase in males up to the age of 20 year. The variation observed in body composition over time closely reflect the development of skeletal muscle. In fact, previous studies (Kanehisa et al., 1994; Neu et al., 2002; Wells et al., 2012) reported that girls displayed cross section areas of different muscle groups similar to those of males during early childhood, a gender separation in muscle quantity taking place after the age of 12–13 year. Thus, our observation that, in obese boys over 12 year, the absolute power was significantly higher than that of obese girls of the same age, appears to be supported by the finding of a correspondingly higher amount of FFM. Therefore, in agreement with previous observations in obese children and adolescents (Duche et al., 2002; Maffiuletti et al., 2004; Sartorio et al., 2006) and adults (Sartorio et al., 2001, 2003b,c, 2004c, 2005; Lafortuna et al., 2002, 2004, 2005), the normalization of absolute power values for FFM eliminated all the observed gender-related difference in children and adolescents (Figures 2G,H) of the present investigation, and the difference in total amount of FFM appeared to be a main determinant of the difference in absolute power observed as a function of gender and age.

However, the power output normalized for BM was significantly higher in boys aged 9–18 years, which suggests that girls with a higher degree of obesity or FM being more limited in anaerobic motor performance in tasks which, like the climbing exercise used in the present study, entail a vertical displacement of the body. This finding suggests that severely obese subjects had poorer performance in all tasks requiring propulsion or lifting of the body mass, and entailing a relevant anaerobic performance (Deforche et al., 2003; Ceschia et al., 2016).

After 3-week BWRP, time of SCT execution decreased in both genders (Figures 2A,B). As a consequence, estimated lower limb muscle power significantly increased in absolute values (Figures 2C,D) and when normalized to the BM (Figures 2E,F) or FFM (Figures 2G,H) in both genders, as previously observed (Sartorio et al., 2001, 2003b,c, 2004a,c, 2005, 2006; Lafortuna et al., 2002, 2004, 2005; Maffiuletti et al., 2004).

The increase in absolute lower limb muscle power, also when normalized for BM or FFM, might be due to the fact that the weight management program induced an important reduction in BM, especially FM, and a maintenance of FFM. The lower limb muscle hypertrophy, in obese children and adolescents, is mainly related to continuous “strength training” associated to excessive body mass, which could be the main factor influencing lower limb muscle power (Maffiuletti et al., 2007).

This finding suggests that functional activities of daily living, as well as muscle function, are easily improved in obese individuals of all ages by a short term intervention entailing moderate energy restriction and physical activity. Particularly, this study shows that structured physical activity programs should be essential to improve physical capacities and quality of life of obese subjects. As a result, given the alarming prevalence of obese children and adolescents and the lower fitness levels, it is very important to identify children that are likely to develop low level of physical fitness to adopt appropriate measures to counteract these deficiencies.

## Conclusion

Our data show that a 3-week multidisciplinary BWRP induces a significant decrease in BM and FM, a maintenance in FFM and a significant improvement of lower limb muscle power both in obese girls and boys. Since obese females showed lower muscle power improvements, probably related to the imbalance between contractile elements and the added inert mass of fat, further additional studies are requested to define physical activity protocols (in the BWRP context) better tailored for this subgroup.

## Data Availability Statement

The datasets generated for this study are available on request to the corresponding author.

## Ethics Statement

The studies involving human participants were reviewed and approved by Italian Institute for Auxology (Milan, Italy). Written informed consent to participate in this study was provided by the participants’ legal guardian/next of kin.

## Author Contributions

SL and AS conceived and designed research. GT, RD, and AD conducted experiments. SL and GB analyzed the data. SL, GB, and AS wrote the manuscript. All authors read and approved the manuscript.

## Conflict of Interest

The authors declare that the research was conducted in the absence of any commercial or financial relationships that could be construed as a potential conflict of interest.
